# Development and internal validation of machine learning models for predicting prediabetes in China

**DOI:** 10.1371/journal.pone.0361704

**Published:** 2026-11-04

**Authors:** Zhengxin Jiang, Wen Qian, Haifang Yang, Yifang Luo, Wen Peng, Qiong Wu, Xiaoxing Wei

**Affiliations:** 1 Department of Physical Education, Qinghai University, Xining, People’s Republic of China; 2 Medical College, Qinghai University, Xining, People’s Republic of China; Tecnologico de Monterrey, MEXICO

## Abstract

**Background:**

Diabetes imposes a growing health burden among Chinese adults, necessitating scalable tools for early identification of high-risk individuals. We aimed to develop and internally validate survival machine-learning models for prediabetes risk stratification using routinely available clinical indicators.

**Methods:**

This retrospective cohort study included 77,478 Chinese adults from the Dryad repository (2010–2016). Candidate predictors were selected via LASSO regression and random survival forests (RSF). Six survival models, Cox proportional hazards (Cox-PH), CoxBoost, DeepSurv, gradient boosting machine (GBM), random survival forests (RSF), and survival tree, were developed and evaluated using the concordance index (C-index), time-dependent ROC curves, calibration curves, and decision curve analysis (DCA). The best-performing model was deployed as a web-based application.

**Results:**

Eight predictors were consistently selected across methods: age, BMI, fasting plasma glucose, total cholesterol, triglycerides, HDL-C, LDL-C, and creatinine clearance rate. In the test set, DeepSurv and GBM achieved comparable discrimination (C-index: 0.756 vs. 0.751; adjusted P = 0.694). GBM was preferred due to better calibration and greater net benefit across clinically relevant thresholds, leading to its selection as the candidate model.

**Conclusion:**

The GBM-based prediabetes risk model, built on eight inexpensive and routinely available clinical measures, showed promising internal discrimination and calibration in a large Chinese cohort. An interactive web tool was developed as a research prototype to facilitate future external validation and early risk identification. External validation in independent populations is warranted before clinical use.

## 1. Introduction

Diabetes is a complex multisystem metabolic disease in which blood sugar levels are persistently increased above the normal level, which can lead to acute and chronic complications [1,2]. Overall, diabetes is considered the third leading contributor to the global disease burden (GDB), with 529 million diabetics worldwide in 2021, corresponding to an age-standardized prevalence rate of 6.1%. Moreover, it is estimated that by 2050, there will be 1.31 billion diabetics worldwide [3]. Notably, diabetes is characterized by hidden onset with little early signs and symptoms, and if left untreated, it can lead to both acute and chronic complications [4]. Prediabetes is an intermediate stage between normal glycemia and overt diabetes [5,6]. Interventions during prediabetes period, normally 9–12 years, could prevent the development of diabetes and its complications [6,7]. Therefore, early prediction, especially screening individuals with prediabetes from healthy population, is of great significance.

Machine learning is an algorithm of artificial intelligence (AI) that can be used to build models of chronic disease prediction, including diabetes and prediabetes, by mining large amounts of medical data [8,9]. However, despite numerous diabetes risk scoring methods, prediabetes remains underappreciated in clinical practice [10]. Moreover, there are limitations in the existing research: first, many models treat prediabetes as a cross-sectional dichotomous outcome measure and do not take into account different lengths of follow-up. Second, many parameters of model are not directly derived from clinical practice, lacking value of application. Third, the limited sample size affected the generalization of these constructed models. China now has the highest number of people living with diabetes worldwide, as well as a larger population of prediabetes individuals [11]. The efficient detection of prediabetes among the general population is of critical importance to arresting the accelerating upward trend in diabetes prevalence in China [12]. Accordingly, we developed and internally validated six survival-based machine learning models for prediabetes risk prediction in a large Chinese cohort, using routinely available clinical indicators.

## 2. Materials and methods

### 2.1 Data sources

Data were obtained from the Dryad repository (https://datadryad.org/stash). The data were accessed for research purposes on May 1, 2025. All data were fully de-identified, and the authors did not have access to information that could identify individual participants during or after data collection. The secondary analysis was performed in accordance with established protocols and regulations [13]. Data on diabetic status and metabolic indicators were collected, extracted and subsequently shared via the Dryad database [14]. The study enrolled participants from 32 districts across 11 cities in China, including Shanghai, Beijing, and Shenzhen. Data comprised medical examination records for all participants spanning 2010–2016. As this was a reanalysis of publicly available, de-identified data, no additional ethical approval was required.

### 2.2 Inclusion and exclusion criteria

In the original study by Chen et al. [14], all enrolled participants were aged over 20 years and had undergone at least two medical examinations during follow-up, with a total of 211,833 participants enrolled.

We further refined the participant cohort by applying the following exclusion criteria (Fig 1):

(1) 26,247 participants with prediabetes at baseline were excluded.
(2) Participants with missing data for any of the following variables were excluded: systolic blood pressure (SBP) (n = 16), diastolic blood pressure (DBP) (n = 1), total cholesterol (TC) (n = 4,238), triglycerides (TG) (n = 32), high-density lipoprotein cholesterol (HDL-C) (n = 79,618), low-density lipoprotein cholesterol (LDL-C) (n = 164), alanine aminotransferase (ALT) (n = 373), serum urea nitrogen (SUN) (n = 2,251), and creatinine clearance rate (CCr) (n = 21).
(3) Participants with extreme values for age (n = 917), body mass index (BMI) (n = 1,127), systolic blood pressure (SBP) (n = 1,201), diastolic blood pressure (DBP) (n = 958), baseline fasting plasma glucose (FPG) (n = 1,302), total cholesterol (TC) (n = 1,264), triglycerides (TG) (n = 4,877), high-density lipoprotein cholesterol (HDL-C) (n = 1,140), low-density lipoprotein cholesterol (LDL-C) (n = 1,407), alanine aminotransferase (ALT) (n = 5,916), serum urea nitrogen (SUN) (n = 1,063), and creatinine clearance rate (CCr) (n = 222) were also excluded.

**Fig 1:**
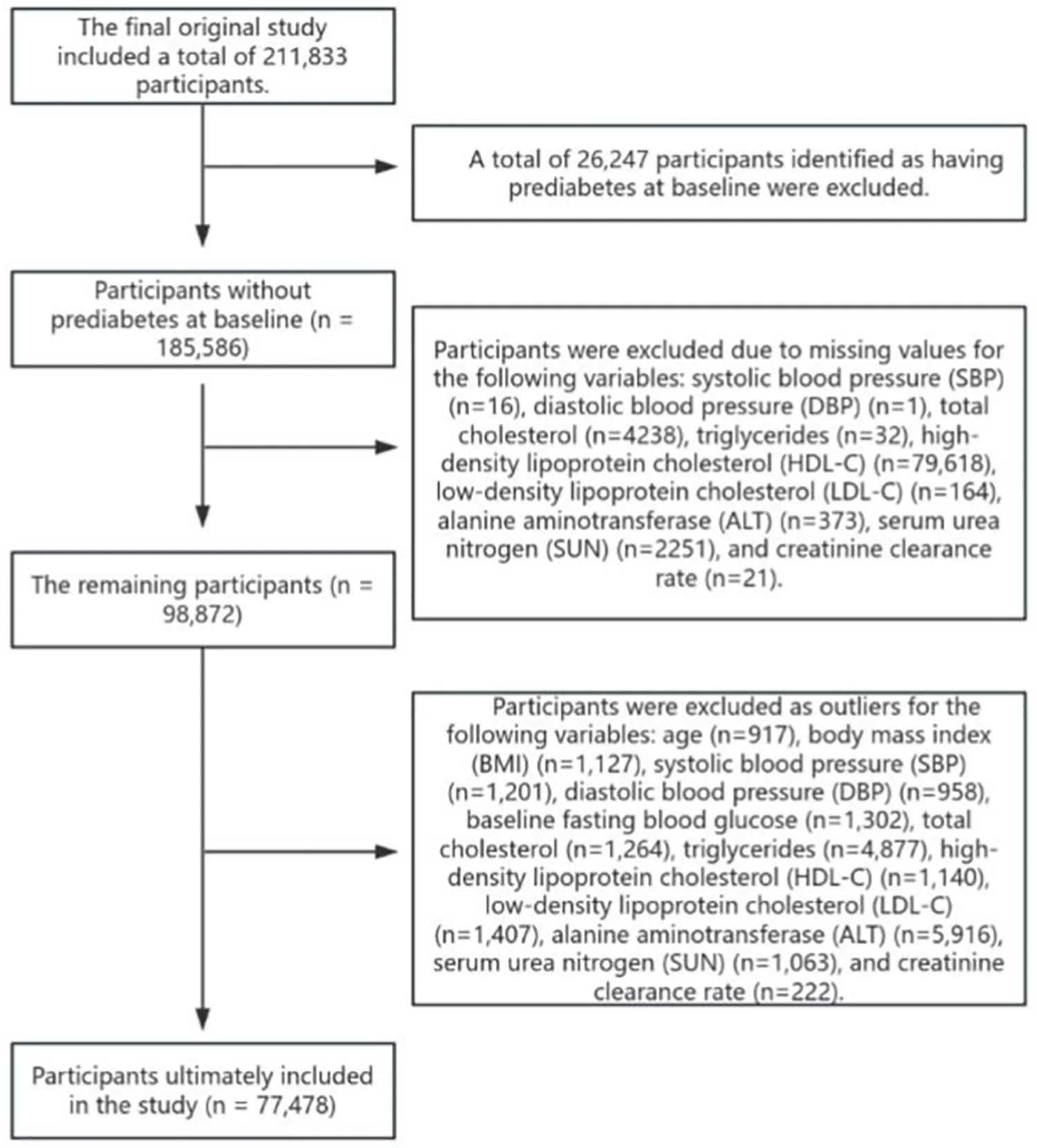
Flowchart of participant selection from the original cohort. Participants with prediabetes at baseline were excluded first. Those with missing values for any key indicator were then excluded, followed by exclusion of extreme values for multiple indices. A final sample of 77,478 participants was included in the analysis.

After applying these criteria, 77,478 participants were included in the final cohort.

#### 2.2.1 Data collection and definitions.

The primary outcomes were incident prediabetes and follow-up duration (survival time). Prediabetes was difined as fasting plasma glucose (FPG) between 5.6 and 6.9 mmol/L (100–125 mg/dL) [6,15], or a self-reported diagnosis of diabetes during follow-up (as individuals with diabetes were considered to have passed through the prediabetes stage). Follow-up time was calculated from the baseline examination to the occurrence of the first of these two events.

Covariates included age, height, weight, gender, body mass index (BMI), systolic blood pressure (SBP), diastolic blood pressure (DBP), fasting plasma glucose (FPG), total cholesterol (TC), triglycerides (TG), high-density lipoprotein cholesterol (HDL-C), low-density lipoprotein cholesterol (LDL-C), alanine aminotransferase (ALT), blood urea nitrogen (BUN), creatinine clearance Rate (CCr), and family history. All variables were collected by trained research personnel [16]. Gender and family history were categorical; all others were continuous. BMI was calculated as weight (kg)/[height (m)]^2^. All remaining variables were considered potential independent variables for subsequent feature selection and model construction.

### 2.3 Data preprocessing and stratification

Observations with missing data for any variable were excluded from the analysis (complete-case analysis). To eliminate the effects of different units of measurement, all continuous variables were standardized using Z-scores prior to modeling [17]. The final cohort of 77,478 participants was randomly split into training and test sets in a 7:3 ratio, yielding 54,234 and 23,244 individuals, respectively. Feature selection, hyperparameter tuning, and model fitting were performed exclusively on the training set; the test set was used only once for final internal validation.

### 2.4 Feature selection

Among the 14 recorded variables were, feature selection was performed using least absolute shrinkage and selection operator (LASSO) regression and the random survival forest (RSF) algorithm, with clinical relevance guiding feature inclusion. Variable importance (VIM) was calculated and visualized for all features. LASSO regression applies L1 regularization to regression coefficients for feature selection and model simplification [18]. RSF extends random forest to survival data, integrating survival analysis with ensemble learning, and demonstrates strong predictive performance and robustness for predicting survival time or probability [19].

### 2.5 Model development

Using the selected variables, we developed six risk prediction models for prediabetes: Cox Proportional Hazards Model (Cox-PH), CoxBoost, DeepSurv, Gradient Boosting Machine (GBM), Random Survival Forests (RSF), and Survival Tree (ST). Cox-PH provided a comprehensible proportional-hazards baseline; CoxBoost incorporated regularized boosting within a proportional-hazards framework; survival trees offered interpretable nonparametric segmentation; RSF captured nonlinearities and interactions via ensemble survival trees; GBM characterized additive nonlinear risk functions through iterative boosting; and DeepSurv served as a neural-network adaptation of the Cox model. This suite enabled comparison of semi-parametric, tree-based ensemble, and neural survival approaches while maintaining time-to-event data structure. Hyperparameter tuning and optimization were performed for each model [20].

### 2.6 Hyperparameter tuning

Grid search was employed to optimize hyperparameters for each model [21], using the training set only and a random seed of 1995. For the survival tree, maximum depth was explored from 1 to 30 using a five-point grid and five-fold cross-validation. For GBM, the number of trees (1–100) and shrinkage parameter (0–1) were assessed on a 5 x 5 grid via three-fold cross-validation; the selected model used 100 trees with a shrinkage of 0.25. For CoxBoost, step count (5–30) and penalty (0–1) were evaluated on a 2 x 2 grid via three-fold cross-validation. For RSF, potential node sizes of 10, 30, 50, 70, and 90 were assessed, starting with mtry = 5 and 200 trees. DeepSurv used a feed-forward neural Cox model with a single hidden layer; dropout, weight decay, learning rate, and hidden node count were optimized via a two-tier grid with three-fold cross-validation. The final DeepSurv configuration used a single hidden node, hard-sigmoid activation, stochastic gradient descent, batch normalization, 10 epochs, and boundary values of 1 for dropout, weight decay, and learning rate. After tuning, each selected specification was refit to the full training set and evaluated once on the held-out test set. No separate third validation cohort was used. Cross-validation estimates were used solely for within-algorithm hyperparameter selection; all between-model comparisons were based on the same held-out test set.

### 2.7 Model performance assessment and validation

Model performance was assessed using Harrell's C-index, time-dependent ROC AUC at 3, 4, and 5 years, calibration curves, and decision curve analysis (DCA). The C-index and time-dependent AUC both measure discrimination but are not synonymous: the C-index summarizes concordance over observed follow-up, whereas time-dependent AUC assesses discrimination at a specific time point. Calibration curves evaluated agreement between predicted and observed risks. DCA quantified net benefit across clinically plausible threshold probabilities.

Pairwise model comparisons were conducted on the test set using paired bootstrap differences for C-index (1,000 resamples) and paired DeLong tests for fixed-horizon AUC, with Holm adjustment for multiple comparisons. Model selection followed a tiered approach: models had to demonstrate competitive test-set discrimination first; when discrimination was statistically indistinguishable, calibration and DCA determined the preferred model based on clinical decision utility [22,23].

### 2.8 Statistical analysis

Statistical analyses were conducted using the R version 4.3.0 (R foundation for statistical computing, Vienna, Austria). Continuous variables were presented as means ± standard deviation (SD) if normally distribution, with group comparisons using Student's *t*-test. Otherwise, median and interquartile range (IQR) were reported, with group comparisons using the Mann-Whitney U-test. Categorical variables were presented as frequencies and percentages (n [%]) and compared using the chi-squared test or Fisher's exact test, as appropriate. All continuous variables were Z-score standardized before analysis. Feature selection was performed using LASSO regression and RSF. Test-set C-index values and 95% confidence intervals were estimated using 1,000 bootstrap resamples. Pairwise C-index differences were evaluated using paired bootstrap resampling of the same participants, with Holm adjustment across the 15 model comparisons. For 3-, 4-, and 5-year binary endpoints, paired DeLong tests was used to compare ROC AUCs. A two-sided α of 0.05 was considered statistically significant.

## 3. Results

### 3.1 Baseline characteristics

After applying the exclusion criteria, 77,478 participants were included in the analysis (Fig 1; Table 1). By the end of follow-up, 9,907 participants had developed prediabetes, while 67,571 remained non-prediabetic. The mean age was 42.6 ± 11.9 years, with a balanced gender distribution (52.2% male, 47.8% female).

**Table 1:** Characteristics of participants at baseline.

Variables	Total (n=77478)	No prediabetes (n=67571)	Developed prediabetes (n=9907)	P -value*
Age (years)	42.6 ± 11.9	41.7 ± 11.5	48.5 ± 12.8	<0.001
BMI (kg/m^2^)	23.1 ± 3.2	22.9 ± 3.2	24.5 ± 3.3	<0.001
SBP (mmHg)	116.0 (106.0, 128.0)	116.0 (106.0, 127.0)	124.0 (113.0, 135.0)	<0.001
DBP (mmHg)	73.0 (66.0, 80.0)	72.0 (66.0, 80.0)	77.0 (70.0, 85.0)	<0.001
FPG (mmol/L)	4.8 (4.5, 5.1)	4.8 (4.5, 5.1)	5.1 (4.8, 5.4)	<0.001
TC (mmol/L)	4.7 (4.1, 5.3)	4.6 (4.1, 5.2)	4.8 (4.3, 5.5)	<0.001
TG (mmol/L)	1.1 (0.7, 1.6)	1.0 (0.7, 1.5)	1.3 (0.9, 2.0)	<0.001
HDL-C (mmol/L)	1.4 (1.2, 1.6)	1.4 (1.2, 1.6)	1.3 (1.1, 1.5)	<0.001
LDL-C (mmol/L)	2.7 (2.3, 3.1)	2.7 (2.3, 3.1)	2.8 (2.4, 3.2)	<0.001
ALT (U/L)	17.8 (12.8, 26.9)	17.1 (12.4, 26.0)	21.0 (15.0, 32.0)	<0.001
BUN (mmol/L)	4.5 (3.8, 5.3)	4.5 (3.8, 5.3)	4.7 (4.0, 5.5)	<0.001
CCr (µmol/L)	69.0 (57.5, 80.8)	68.1 (57.0, 80.3)	72.7 (61.0, 83.0)	<0.001
Gender (%)				<0.001
male	40448 (52.2)	34232 (50.7)	6216 (62.7)	
female	37030 (47.8)	33339 (49.3)	3691 (37.3)	
Family history of diabetes (%)				0.017
No	75715 (97.7)	66067 (97.8)	9648 (97.4)	
Yes	1763 (2.3)	1504 (2.2)	259 (2.6)	

Abbreviations: BMI, body mass index; SBP, systolic blood pressure; DBP, diastolic blood pressure; FPG, fasting plasma glucose; TC, total cholesterol; HDL-C, high-density lipoprotein cholesterol; LDL-C, low-density lipoprotein cholesterol; ALT, alanine aminotransferase; BUN, blood urea nitrogen; CCr, creatinine clearance rate. *P-values were derived from Student's t-test, Mann-Whitney U-test, or chi-squared test, as appropriate.

Compared with non-prediabetic participants, those who developed prediabetes had significantly higher baseline levels of FPG, BMI, systolic blood pressure, diastolic blood pressure, TC, TG, LDL-C, ALT, serum urea nitrogen, and CCr (all P < 0.01). By contrast, and significantly lower HDL-C levels (1.3 vs. 1.4 mmol/L; P < 0.05). Full baseline characteristics are summarized in Table 1.

### 3.2 Feature selection

Among the 16 variables analyzed, LASSO regression selected 13 variables, including age, BMI, SBP, DBP, FPG, TC, TG, HDL-C, LDL-C, ALT, SUN, CCr, and family history of diabetes. RSF ranked variable importance and identified the top 8 predictors: FPG, age, BMI, TC, CCr, HDL-C, LDL-C, and TG (Fig 2). The intersection of the variables selected by both models, age, BMI, FPG, TC, TG, HDL-C, LDL-C, and CCr, were used for subsequent model construction. The selection indicates predictive contribution within this dataset and should not be interpreted as evidence of causality.

**Fig 2:**
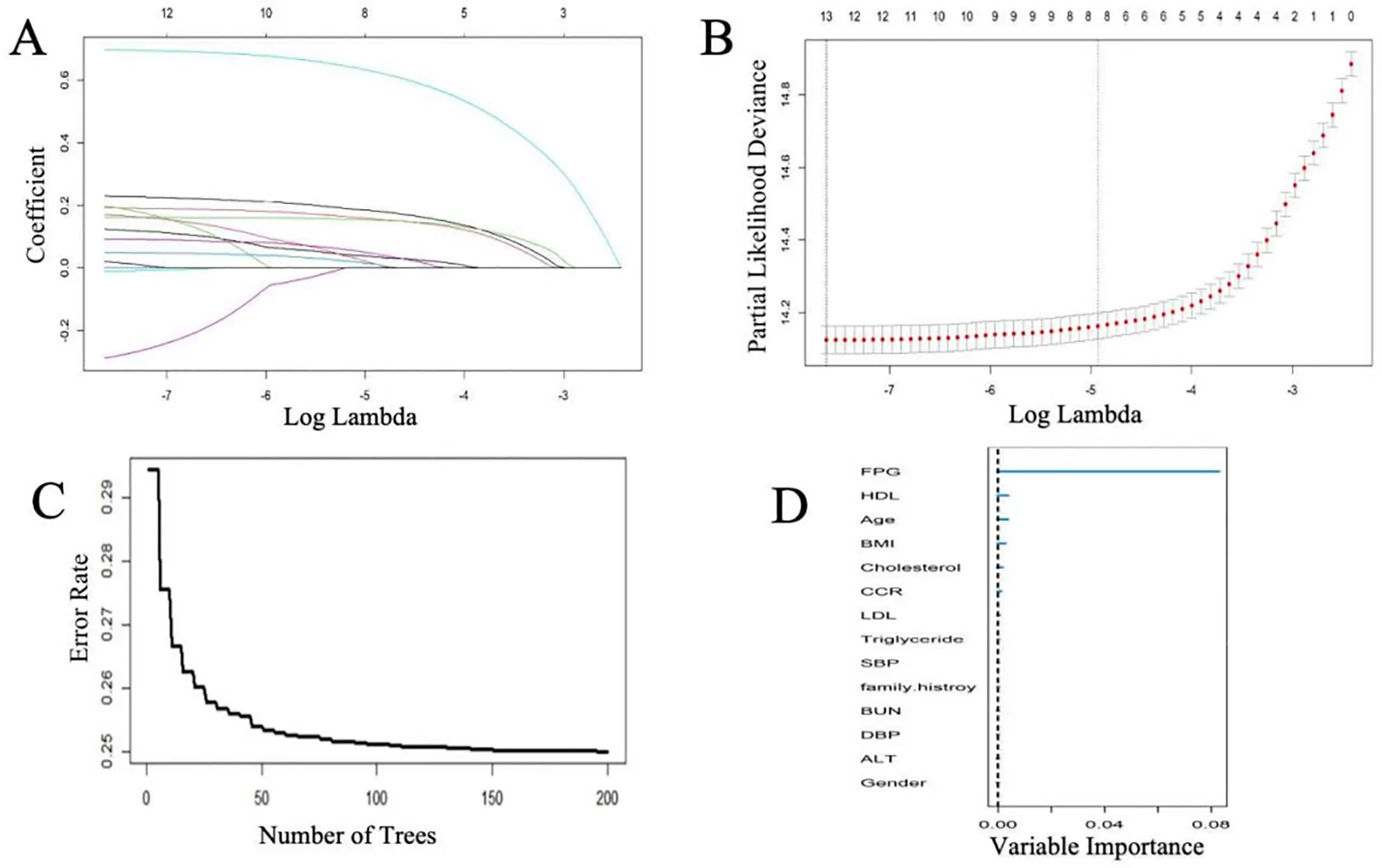
Variable selection using LASSO regression and random survival forests (RSF). A, LASSO coefficient paths for each predictor. B, Cross-validated partial-likelihood deviance used to select the optimal lambda (lambda.min). C, RSF out-of-bag (OOB) error rate across 200 trees. D, Permutation-based variable importance ranking. ALT, alanine aminotransferase; BMI, body mass index; BUN, blood urea nitrogen; CCr, creatinine clearance rate; DBP, diastolic blood pressure; FPG, fasting plasma glucose; HDL-C, high-density lipoprotein cholesterol; LDL-C, low-density lipoprotein cholesterol; SBP, systolic blood pressure.

### 3.3 Model performance evaluation

#### 3.3.1 Discrimination (C-index).

Table 2 presents the C-index values for all six models in the training and test sets. In the test set, DeepSurv achieved the highest C-index (0.756, 95% CI 0.744–0.763), followed by GBM (0.751, 95% CI 0.742–0.761), Cox-PH (0.749, 95% CI 0.740–0.759), CoxBoost (0.749, 95% CI 0.739–0.758), RSF (0.747, 95% CI 0.737–0.756), and ST (0.710, 95% CI 0.700–0.720). The difference between DeepSurv and GBM was not statistically significant (difference 0.0041, 95% CI 0.0013 to 0.0054; unadjusted P = 0.256; Holm-adjusted P = 0.694). After Holm adjustment, DeepSurv outperformed RSF (adjusted P = 0.011), whereas ST performed significantly worse than all other models (all adjusted P < 0.0005). RSF exhibited marked optimism, with a training C-index of 0.822 but a test C-index of 0.747, indicating potential overfitting.

**Table 2:** Assessment of the discriminative ability of the six candidate models.

Model	C-index		95% CI
Deep survival model (DeepSurv)	Training	0.756	0.748-0.761
	Testing	0.756	0.744−0.763
Gradient boost machine (GBM)	Training	0.755	0.749-0.761
	Testing	0.751	0.742−0.761
Cox-PH	Training	0.749	0.743-0.755
	Testing	0.749	0.740-0.759
CoxBoost	Training	0.749	0.742−0.755
	Testing	0.749	0.739-0.758
Random survival forest (RSF)	Training	0.822	0.817-0.827
	Testing	0.747	0.737-0.756
Survival tree (ST)	Training	0.708	0.701-0.714
	Testing	0.710	0.700−0.720

#### 3.3.2 Time-dependent ROC analysis.

Time-dependent ROC curves at 3, 4, and 5 years are shown in Fig 3. Across all time points, DeepSurv and GBM consistently showed the highest test-set AUCs, with DeepSurv marginally ahead at 3 and 4 years, and GBM performing similarly at 5 years. For example, at 3 years, DeepSurv achieved a test AUC of 0.778 (95% CI 0.761–0.787), compared with 0.773 (95% CI 0.761–0.786) for GBM. RSF demonstrated strong training AUCs (e.g., 0.838 at 3 years) that were not reproduced in the test set (0.764), further confirming overfitting. ST consistently underperformed relative to the other models (test AUC range 0.696–0.736 across time points). Detailed AUC values with 95% CIs for all models at each time point are provided in the figure legends.

**Fig 3:**
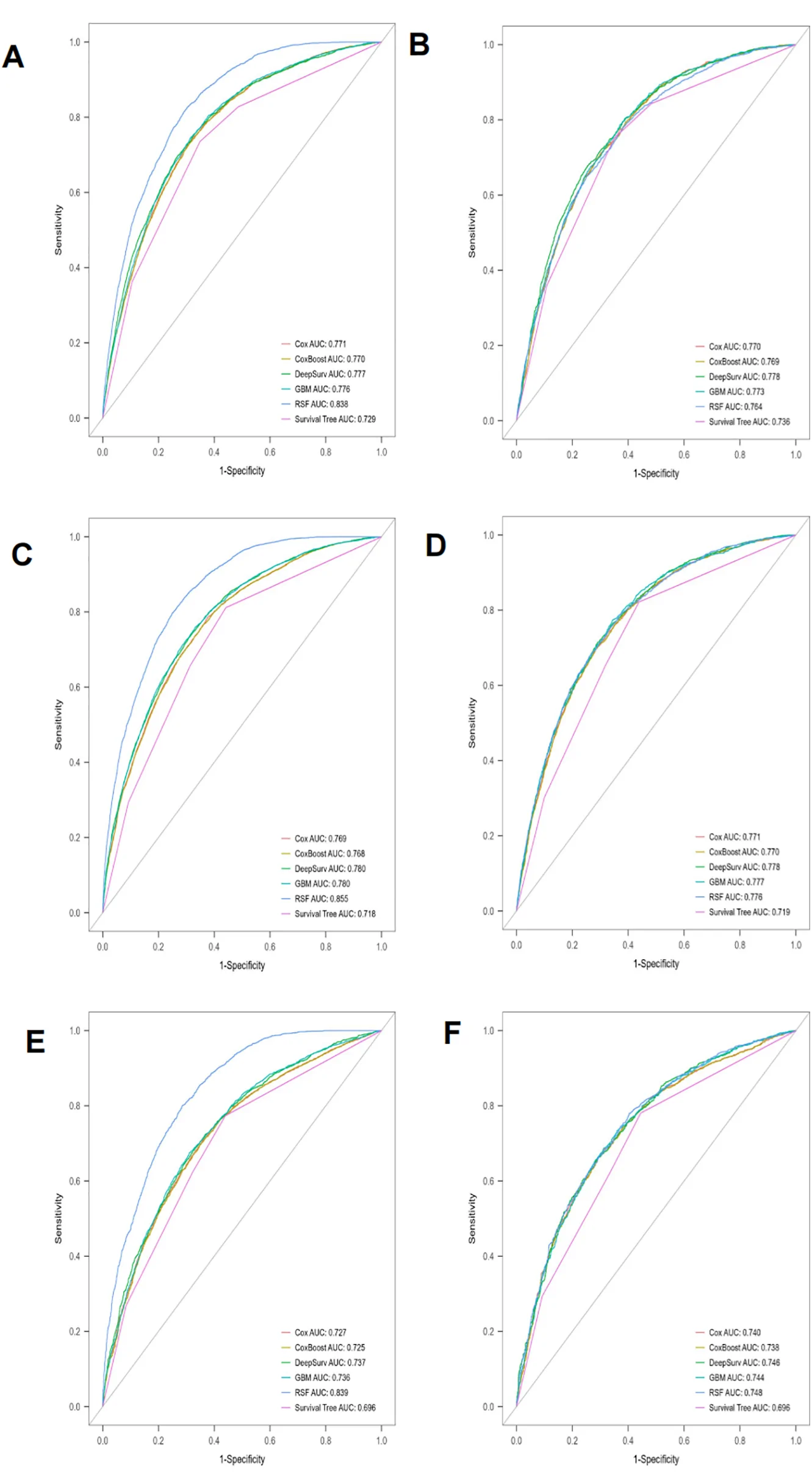
Time-dependent receiver operating characteristic (ROC) curves for the six prediction models. Panels (A, C, E) show the training set results at 3, 4, and 5 years, respectively; panels (B, D, F) show the corresponding test set results. Each panel displays the ROC curves for all six candidate models (Cox-PH, CoxBoost, DeepSurv, GBM, RSF, and Survival Tree) at the specified time horizon.

#### 3.3.3 Calibration.

Calibration curves at 3, 4, and 5 years are presented in Fig 4. GBM and CoxBoost showed the closest agreement between predicted and observed risks across both training and test sets, with calibration curves closely approximating the 45 degree diagonal. In contrast, DeepSurv exhibited visible deviations, particularly in the higher-risk quartiles, suggesting risk overestimation in that range.

**Fig 4:**
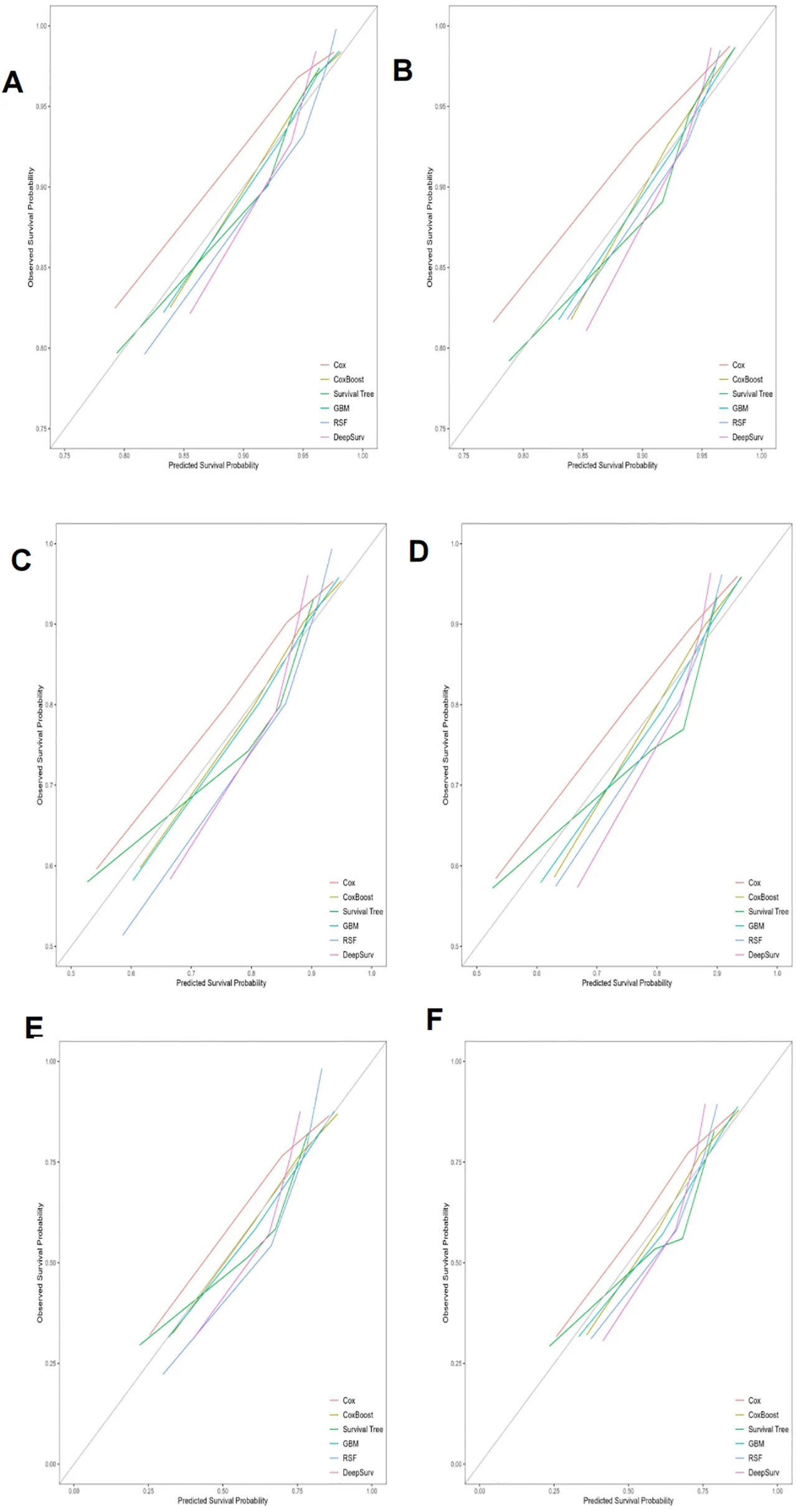
Calibration curves for the six models in the training and test sets. Panels (A, C, E) present the training-set calibration at 3, 4, and 5 years, respectively; panels (B, D, F) present the corresponding test-set results. The diagonal dashed line represents perfect calibration, where predicted risks equal observed event proportions.

#### 3.3.4 Decision curve analysis.

DCA curves for all models are shown in Fig 5. In the test set, GBM consistently provided the highest net benefit across the widest range of clinically relevant threshold probabilities: 2–27% at 3 years, 3–60% at 4 years, and 6–85% at 5 years. This indicates that GBM would yield greater clinical utility than the other models across most decision thresholds.

**Fig 5:**
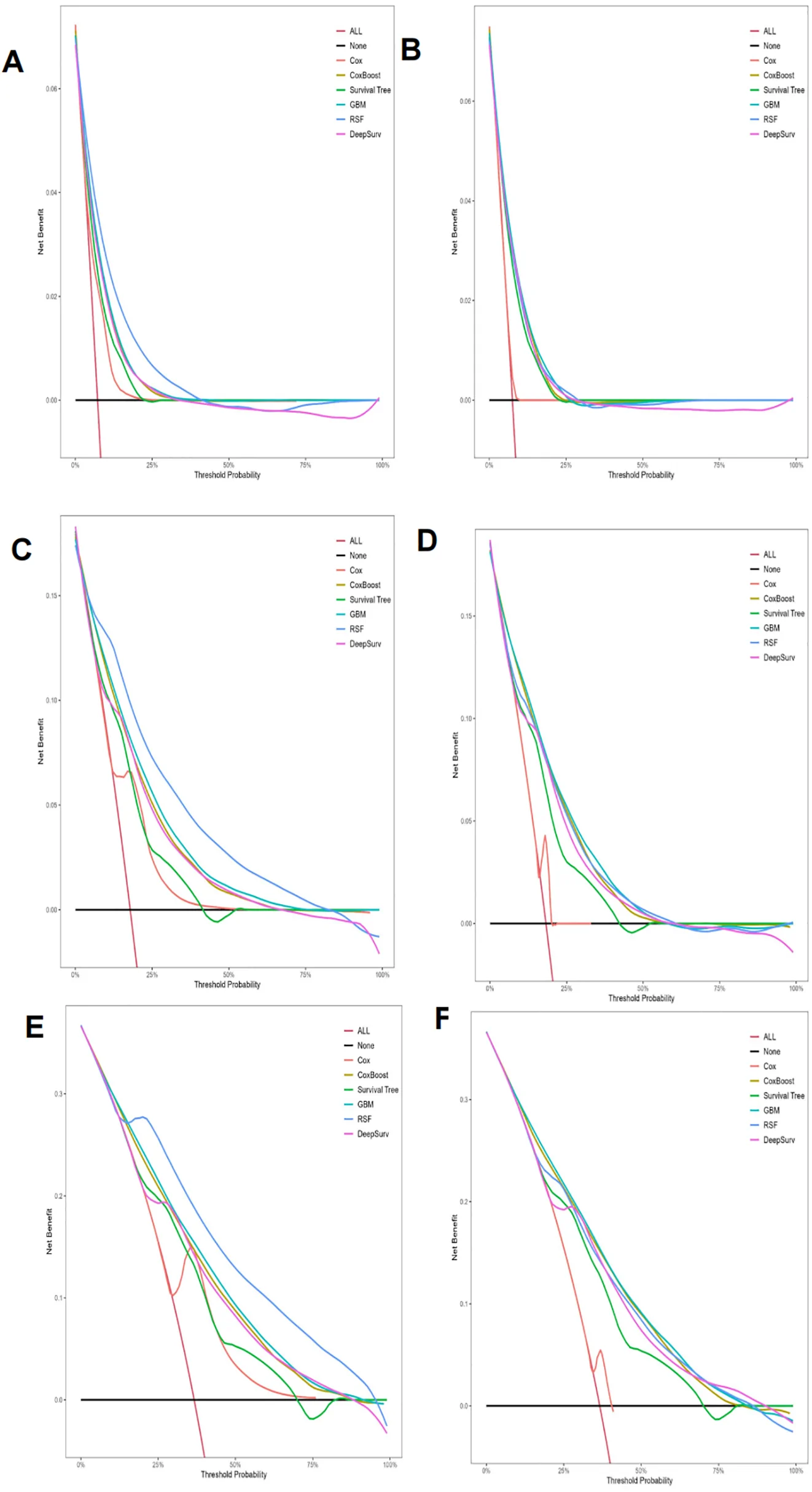
Decision curve analyses (DCA) for the six models in the training and test set. Panels (A, B) show the 3-year DCA, (C, D) the 4-year DCA, and (E, F) the 5-year DCA, with training set results on the left and test set results on the right. The gray horizontal line (y = 0) indicates the net benefit of intervening on no participants; the solid red line indicates the net benefit of intervening on all participants. Curves for each of the six models represent the net benefit of using that model to guide intervention across a range of threshold probabilities. A model's curve lying above both reference lines across a wider threshold range indicates greater clinical utility.

#### 3.3.5 Model selection.

We did not apply an unvalidated numerical weighting across metrics. Instead, we used a hierarchical decision rule: discrimination established a shortlist of competitive models, after which calibration and DCA determined the preferred model. Because the C-index difference between DeepSurv and GBM was small and non-significant, GBM was selected as the best-performing model due to its superior calibration and broader net benefit. This reflects a principled trade-off favoring reliable absolute-risk estimation and decision utility over a negligible numerical advantage in discrimination. This selection is based on internal validation only. Because the test set was a random split of the same cohort, it does not constitute independent external validation.

### 3.4 Visualization of the best-performing model

An interactive web application was developed using the Shiny framework and deployed at https://deliveryridersbehavior.shinyapps.io/gbm_app/. Users can input values for the eight predictors to obtain individual prediabetes risk estimates. Fig 6 illustrates the application output for a hypothetical user aged 42 years old, with a BMI of 23 kg/m^2^, FPG of 4.7 mmol/L, TC of 4.7 mmol/L, TG of 1.3 mmol/L, HDL-C of 1.4 mmol/L, LDL-C of 2.7 mmol/L, and CCr of 70 µmol/L.

**Fig 6:**
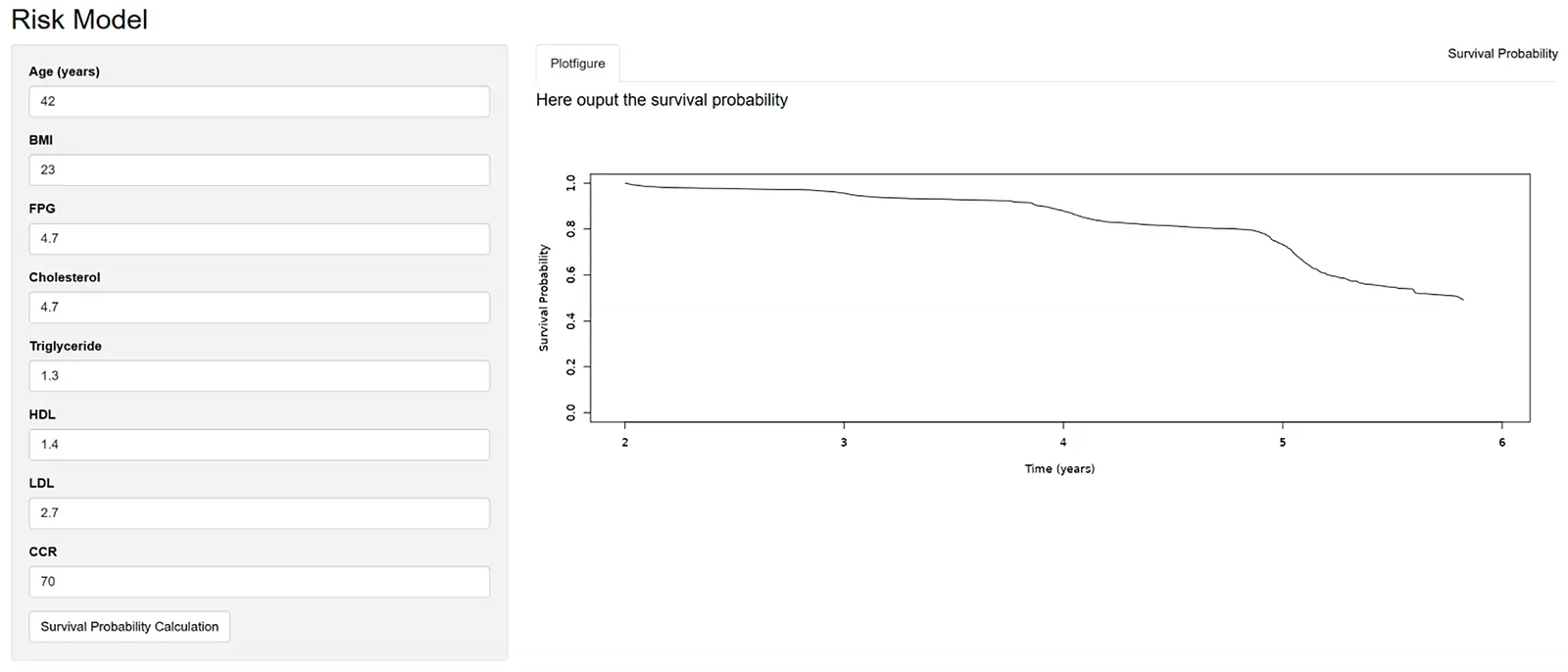
Screenshot of the interactive web-based application for the optimal GBM model. The application outputs estimated prediabetes risk probabilities across follow-up time for an individual user. The example shows results for a representative participant aged 42 years, with BMI 23 kg/m^2^, FPG 4.7 mmol/L, TC 4.7 mmol/L, TG 1.3 mmol/L, HDL-C 1.4 mmol/L, LDL-C 2.7 mmol/L, and CCr 70 µmol/L. The tool is publicly accessible at https://deliveryridersbehavior.shinyapps.io/gbm_app/.

## 4. Discussion

In this large retrospective cohort of 77,478 Chinese adults, we developed and internally validated six survival-based machine learning models for prediabetes risk prediction using eight readily available clinical indicators. Among these, GBM and DeepSurv achieved the highest and statistically comparable discrimination. GBM was ultimately selected because it offered more consistent calibration and greater net benefit across clinically meaningful threshold ranges, a principled trade-off favoring reliable absolute-risk estimation over a negligible numerical advantage in concordance.

The eight predictors identified, age, BMI, FPG, TC, TG, HDL-C, LDL-C, and CCr—are all routinely collected in standard health examinations, supporting the practical deployability of our model. Age and FPG contributed most strongly to prediction, consistent with established epidemiological knowledge. Notably, the concentration of predictive signal in these conventional measures suggests that our model captures established glycemic and demographic trajectories rather than novel complex interactions. This transparency may facilitate clinician acceptance, though it also implies limited added value from more complex feature engineering in this setting.

Compared with previous prediabetes prediction studies, our work offers several methodological and practical advances. First, unlike cross-sectional dichotomous approaches, we modeled time-to-event data, explicitly accounting for varying follow-up durations. This survival framework better reflects the dynamic nature of prediabetes progression and enables risk estimation at clinically relevant time horizons. Second, our sample size (n = 77,478) substantially exceeds that of most published studies in Chinese populations. For instance, Xin et al. developed logistic and decision-tree models in 2,261 rural Chinese participants and reported an AUC of 0.689 [24]; our GBM model achieved a test-set C-index of 0.751, suggesting improved discrimination, though direct comparisons are hampered by differences in predictor sets and outcome definitions. Third, we prioritized clinical utility through DCA, a metric often overlooked in prior reports, rather than relying solely on statistical fit.

Several machine learning-based prediabetes models have been published recently, with widely varying performance. Abbas et al. achieved an AUC of 0.81 in a Qatari cohort using logistic regression and ensemble methods [25], whereas Koopman et al. reported an AUC of 0.740 using logistic regression in U.S. NHANES data [26]. Hische et al. used decision trees in a German sample [27], and Yuk et al. reported an AUC of 0.783 for LightGBM in a Korean population [28]. Notably, machine learning algorithms have not consistently outperformed logistic regression in these comparisons. Our findings echo this pattern: the survival tree performed substantially worse, and the relative advantage of DeepSurv and GBM over Cox-PH was modest. This underscores the importance of comprehensive model evaluation, including calibration and DCA, rather than selecting models solely on discrimination.

Seto et al. observed that gradient boosting methods become more reliable than logistic regression when sample sizes exceed 10,000 [29], which aligns with our observation that GBM maintained stable test-set performance with minimal overfitting. In contrast, RSF showed marked training, test optimism (C-index 0.822 vs. 0.747), highlighting the need for rigorous internal validation even with large samples. Li et al. developed a non-invasive model using tongue imaging combined with genetic algorithm, XGBoost, achieving an AUC of 0.914, but required specialized equipment and did not incorporate time-to-event data, limiting comparability and generalizability [30].

Clinical implications. The web-based application we provide offers a low-barrier entry point for prediabetes risk assessment. By requiring only eight routine laboratory and anthropometric measures, it can be integrated into existing health examination workflows without additional cost or specialized training. However, we emphasize that this tool is intended to support risk awareness and shared decision-making, not as a standalone diagnostic or replacement for clinical judgment.

Limitations. Several limitations merit consideration. First, this was a retrospective reanalysis of an existing urban Chinese cohort; rural populations were not represented, and the findings may not generalize to other geographic or socioeconomic settings. Second, the data originated from routine health examinations, and we relied on FPG-based prediabetes definitions; oral glucose tolerance test results were unavailable, which may have led to misclassification of some cases. Third, calibration curves indicated some risk overestimation in higher-risk quartiles for certain models, and RSF exhibited clear overfitting. Fourth, because the test set was a random split from the same cohort, this internal validation was non-independent and performance may be optimistic. Our web application is thus a research tool for reproducibility and prospective testing rather than a clinically deployed decision aid and requires external validation before clinical use. Fifth, we used listwise deletion for missing data, which may introduce bias if missingness was not completely at random. Finally, the predictors identified represent associations, not causal relationships.

## 5. Conclusion

In this internally validated analysis, GBM was the best-performing candidate among six survival machine-learning models using eight routine clinical measures in a large Chinese cohort. However, because validation was based on a non-independent random split of the same cohort, these findings should be regarded as hypothesis-generating. External temporal or geographic validation is required before the model can be considered for clinical use.

## Abbreviations

AI: Artificial intelligence
AIC: Akaike Information Criterion
ALT: Alanine Aminotransferase
AUC: Area Under the Curve
BMI: Body Mass Index
BUN: Blood Urea Nitrogen
CCr: Creatinine Clearance Rate
C-index: Concordance Index
95% CI: 95% Confidence interval
DBP: Diastolic Blood Pressure
FPG: Fasting Plasma Glucose
GBM: Gradient Boosting Machine
HDL-C: High-Density Lipoprotein Cholesterol
IFG: Impaired Fasting Glucose
IGT: Impaired Glucose Tolerance
LASSO: Least Absolute Shrinkage and Selection Operator
LDL-C: Low-Density Lipoprotein Cholesterol
ML: Machine Learning
ROC: Receiver Operating Characteristic
RSF: Random Survival Forests
SBP: Systolic Blood Pressure
TC: Total Cholesterol
TG: Triglyceride.
